# Supplementary material for: Long non-coding RNA Neat1 regulates adaptive behavioural response to stress in mice
Source: Transl Psychiatry. 2020 May 28;10:171. doi: 10.1038/s41398-020-0854-2 (PMC7256041; doi:10.1038/s41398-020-0854-2)
Supplement: Supplementary file 1 — Supplemental Figures S1-S5 and Tables S1-S3 [file 41398_2020_854_MOESM1_ESM.docx]

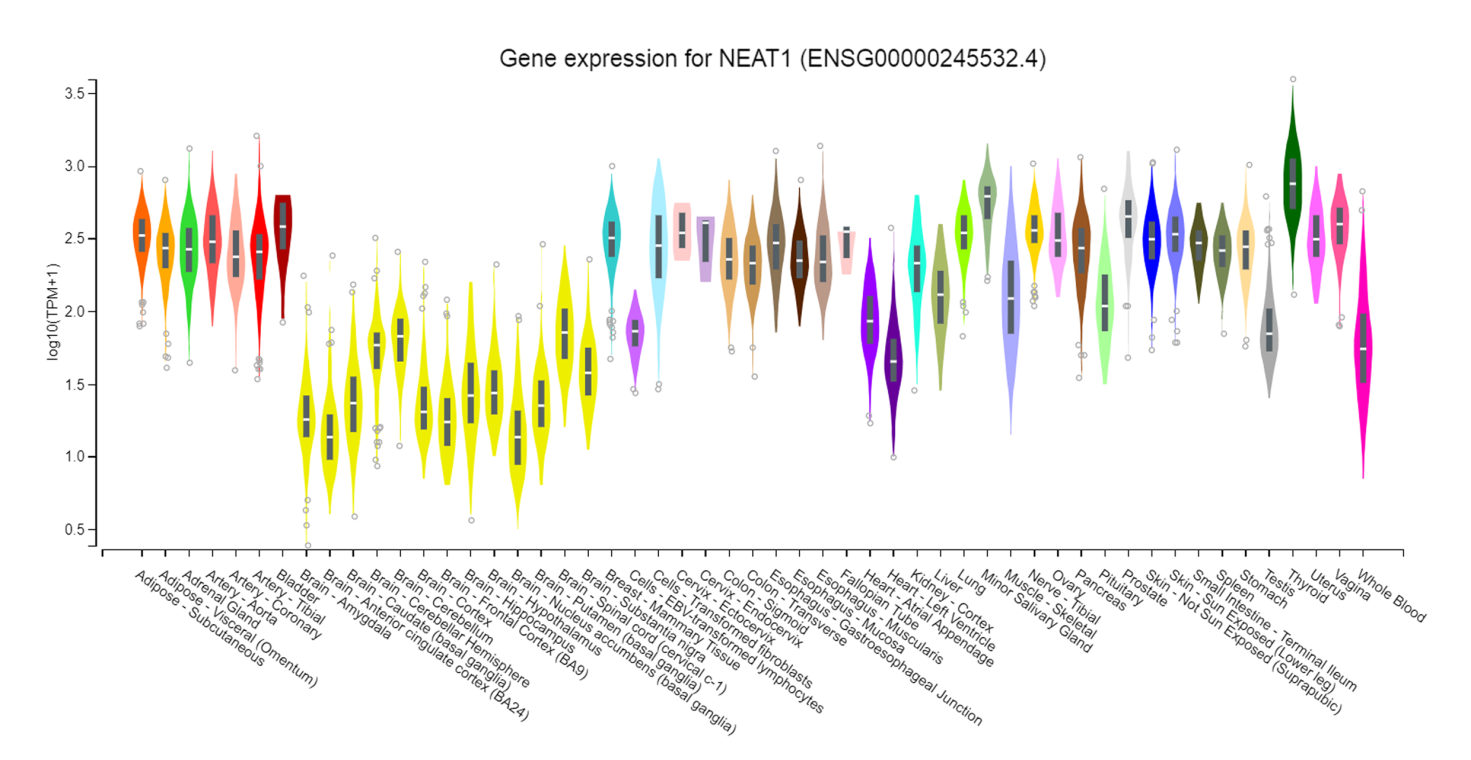


**Fig. S1.** NEAT1 expression in the human organs and tissues according to the Genotype-Tissue Expression (GTEx) database (<https://gtexportal.org/home/>).


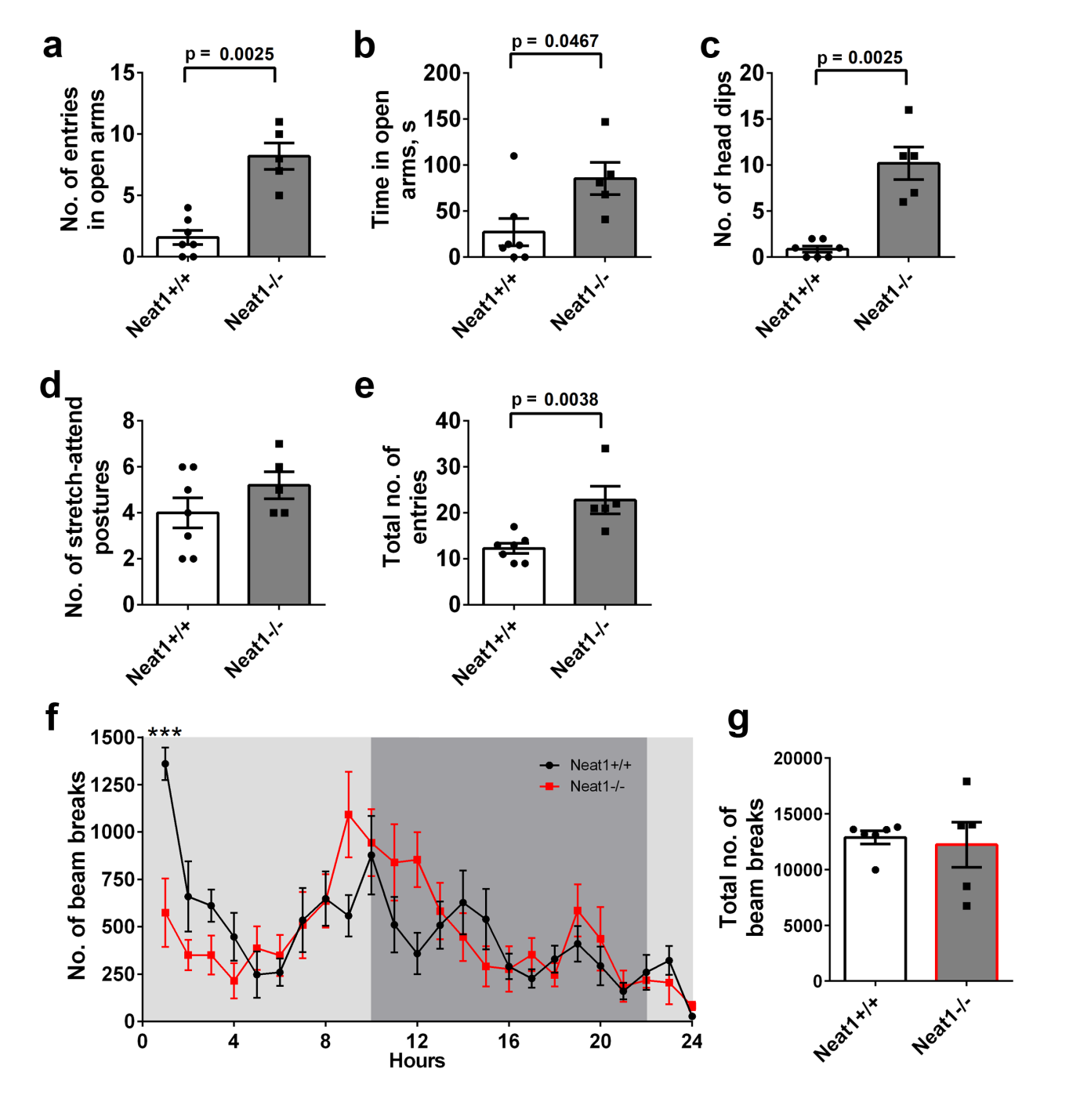


**Fig. S2. The behavioural phenotype of *Neat1^-/-^* mice is preserved during aging.**

Novel cohorts of 18 month-old animals not previously subjected to behavioural testing were used in these tests. **a-e** Performance of *Neat1^-/-^* and *Neat1^+/+^* mice in the elevated plus maze (EPM) test. Increased number of entries (a), increased amount of time spent in the open arms (b), increased number of head dips (c), unaltered number of stretch-attend postures (d) and increased total number of entries (e) for aged *Neat1^-/-^* mice as compared to *Neat1^+/+^* mice (Mann-Whitney *U* test; *Neat1^-/-^* n=5, *Neat1^+/+^* n=7). **f,g** Reduced locomotor activity of *Neat1^-/-^* mice in the Home Cage test during habituation (two-way ANOVA with Holm-Sidak's multiple comparisons test, ***p< 0.001; *Neat1^-/-^* n=5, *Neat1^+/+^* n=6). Number of breaks per hour (f) and total number of breaks over the 24-h period (g) are shown.


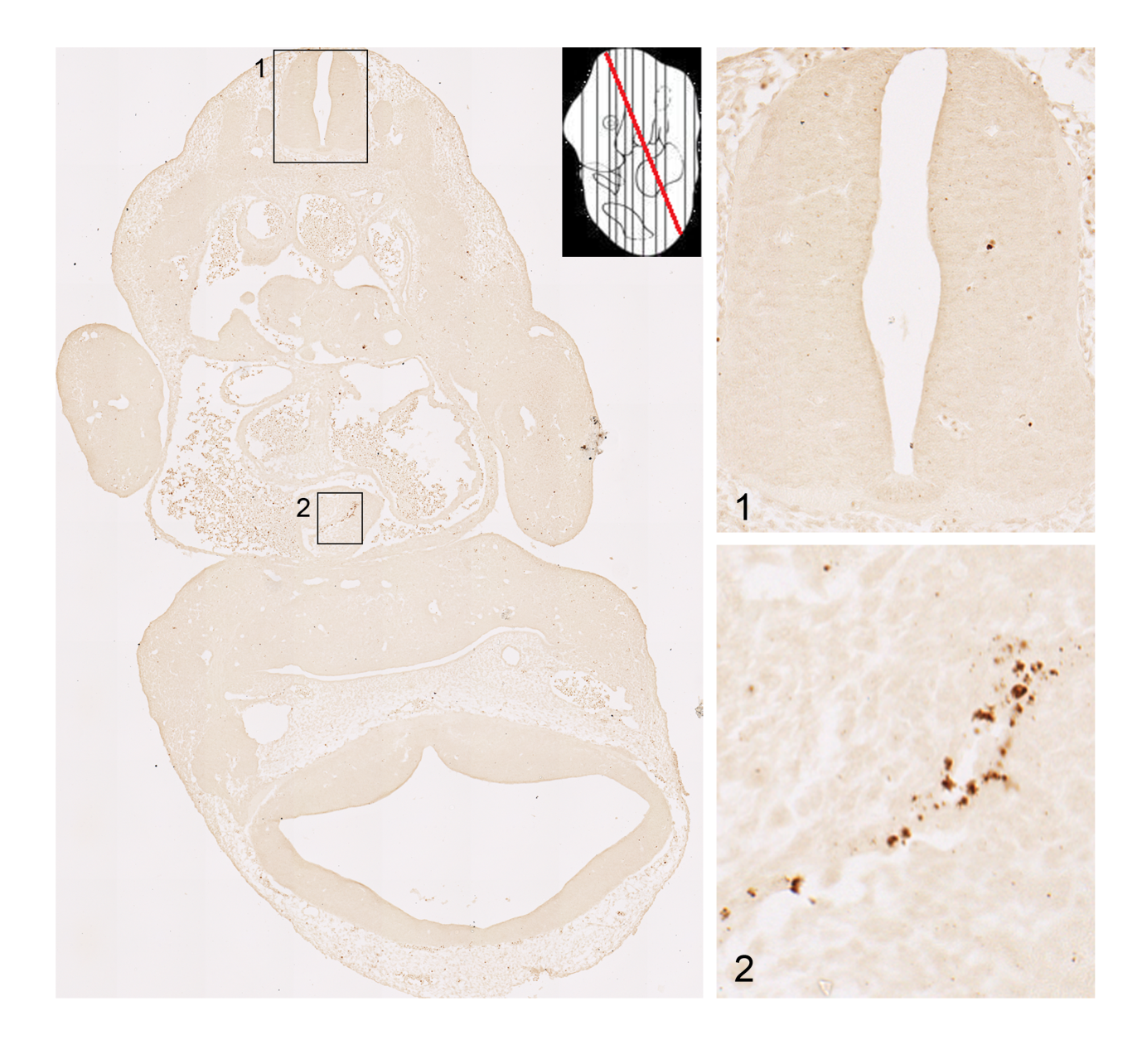


**Fig. S3. Neat1 expression in the E12.5 mouse embryo.**

RNAScope ISH using 5’ fragment Neat1 probe in WT E12.5 mouse embryo. The plane of the section within the embryo is also shown (red line). Numbered insets show two zones at higher magnification: 1 – neural tube, 2 – blood vessel. Note multiple Neat1-positive cells in the blood vessel but not in the neural tube.


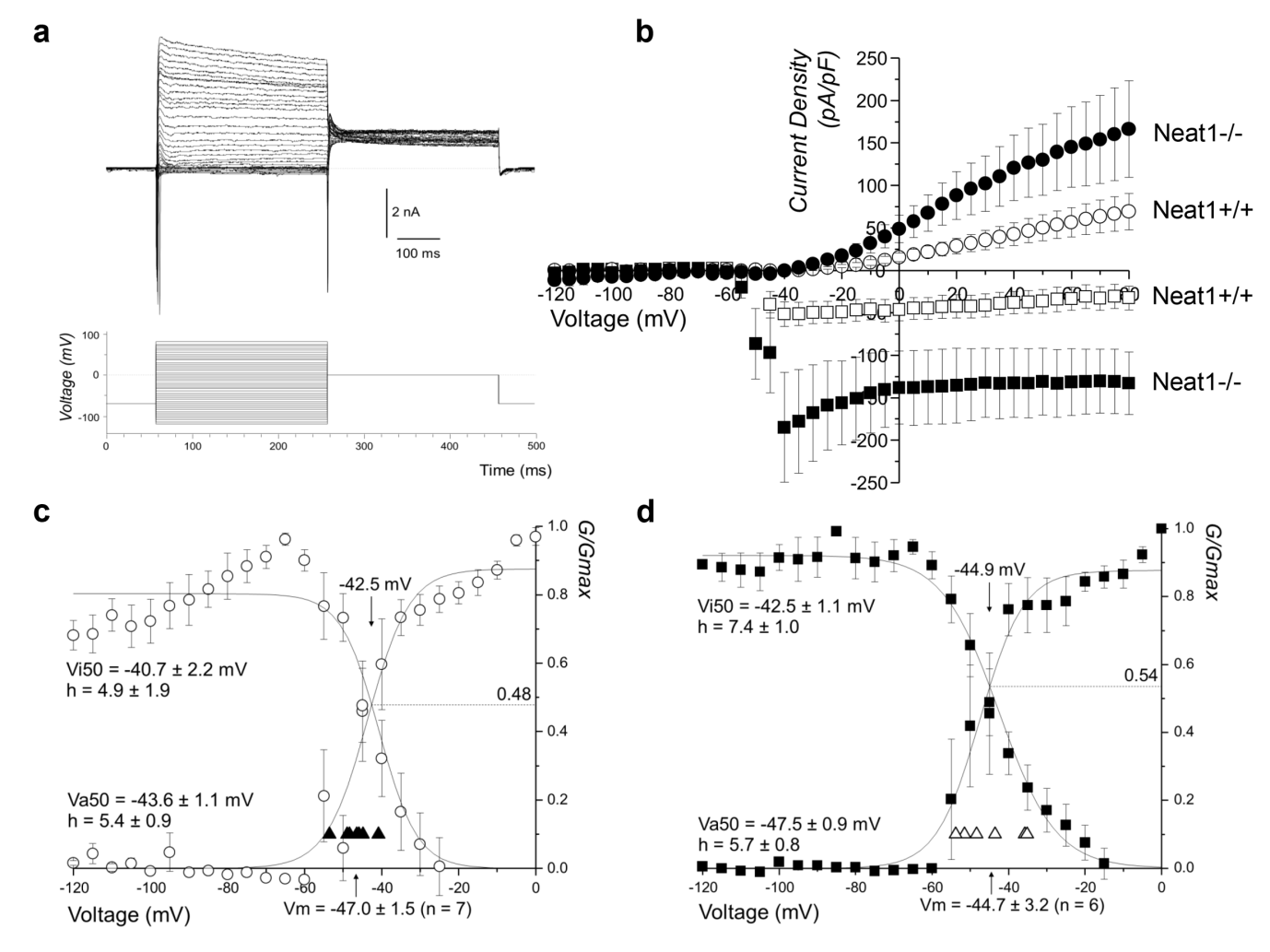


**Fig. S4. The effect of Neat1 loss on transmembrane voltage-gated K+ and Na+ currents in mouse cultured neurons.**

**a** Exemplar families of whole cell currents evoked by the voltage activation/inactivation protocol (as shown at the bottom panel) in mouse neurons. **b** Mean current densities vs. voltage plots derived for voltage-activated Na+ (squares) and K+ currents (circles) derived from traces exemplified in (a). Empty and filled symbols represent the current densities from *Neat1^+/+^* and *Neat1^-/-^* mice, respectively. **c,d** Mean fractional conductance (G/Gmax) plots for voltage activation and inactivation of Na^+^ currents for *Neat1^+/+^* (c) and *Neat1^-/-^* (d) neurons derived from the traces exemplified in (a). On each panel are also shown individual Vm values (triangles) and mean Vm values (arrow on abscissa). Voltages of half-maximal action (Va50) and half-maximal inactivation (Vi50) are also indicated, along with h factors, mean crossing points (downward arrows) number of cells recorded for each group (n).


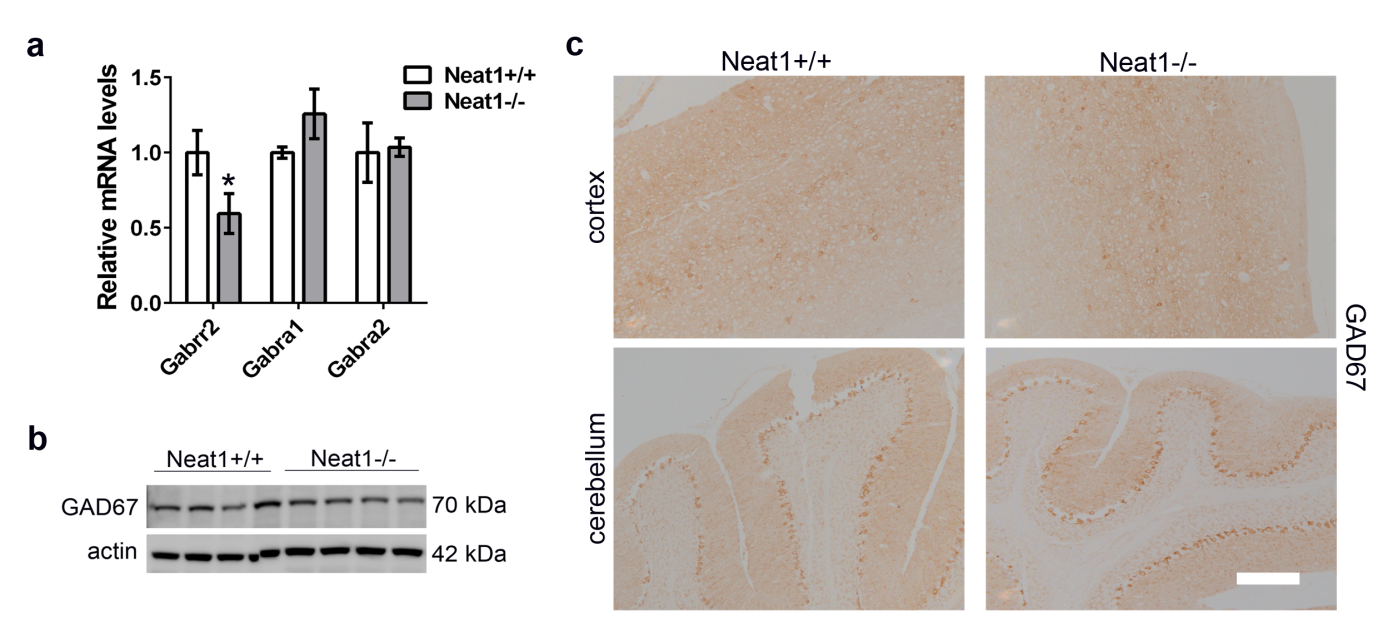


**Fig. S5. Loss of Neat1 does not affect GABAergic neurons in the brain.**

**a** Decreased expression of *Gabrr2* but not *Gabra1* or *Gabra2* in the cortex of *Neat1^-/-^* mice as revealed by qRT-PCR (Mann-Whitney *U* test; *p< 0.05, *Neat1^-/-^* n=4, *Neat1^+/+^* n=4). **b,c** Unaltered Gad67 levels (b) and distribution (c) in the cortex and cerebellum of *Neat1^-/-^* mice as measured by Western blot and immunohistochemistry, respectively. Representative images are shown. Scale bar, 100 µm.

**Table S1.** Proportion (%) of *Neat1^+/+^* (left) and *Neat1^-/-^* (right) mouse hippocampal neurons which demonstrated each of the different types of spontaneous action potentials (sAP type: quiet, attempting or spontaneous).

| Neat1^+/+^ | | | | Neat1^-/-^ | | | |
| --- | --- | --- | --- | --- | --- | --- | --- |
|  | Type of activity | (%) | n |  | Type of activity | (%) | n |
|  |  | 100% | 11 |  |  | 100% | 6 |
| sAP type (%) | Quiet | 18% | 2 | sAP type (%) | Quiet | 0% | 0 |
|  | Attempting | 45% | 5 |  | Attempting | 50% | 3 |
|  | Spontaneous | 36% | 4 |  | Spontaneous | 50% | 3 |

**Table S2.** Proportion (%) of *Neat1^+/+^* (left) and *Neat1^-/-^* (right) mouse hippocampal neurons which demonstrated each of the different types of induced action potentials (iAP type: none, attempting single, single, attempting train, train).

| Neat1^+/+^ | | | | Neat1^-/-^ | | | |
| --- | --- | --- | --- | --- | --- | --- | --- |
| iAP type | Total number of cells | (%) | n | iAP type | Total number of cells | (%) | n |
|  |  | 100% | 10 |  |  | 100% | 5 |
|  | None | 0% | 0 |  | None | 0% | 0 |
|  | Attempting single | 0% | 0 |  | Attempting single | 0% | 0 |
|  | Single | 30% | 3 |  | Single | 0% | 0 |
|  | Attempting train | 30% | 3 |  | Attempting train | 0% | 0 |
|  | Train | 40% | 4 |  | Train | 100% | 5 |

**Table S3.** Comparison of passive and active parameters of *Neat1^+/+^* (left) and *Neat1^-/-^* (right) mouse hippocampal neurons*.* *Significantly different (p < 0.05) from *Neat1^+/+^* mouse neurons. Abbreviations: Membrane potential (Vm); input resistance (Rin), and; whole cell capacitance (Cp); I Na max and I K max are maximal values of sodium (Na^+^) and potassium (K^+^) transmembrane currents.

|  |  | Neat1^+/+^ | | | Neat1^-/-^ | | |
| --- | --- | --- | --- | --- | --- | --- | --- |
|  |  | Mean | SEM | n | Mean | SEM | n |
| Passive | Vm (mV) | -45.6 | 1.6 | 11 | -44.7 | 3.2 | 6 |
|  | Rin (GΩ) | 0.6 | 0.1 | 10 | 0.7 | 0.1 | 5 |
|  | Cp (pF) | 33.5 | 5.9 | 10 | 20.2 | 3.1 | 5 |
| Spike analysis | Threshold (mV) | -38.1 | 1.8 | 10 | -43.2 | 4.0 | 5 |
|  | Overshoot (mV) | 17.0 | 4.3 | 10 | 28.1 | 7.0 | 5 |
|  | After hyperpolarization (mV) | -52.1 | 2.9 | 10 | -61.4 | 5.4 | 5 |
|  | Amplitude (mV) | 69.1 | 6.2 | 10 | 89.6 | 9.9 | 5 |
|  | Depolarization rate (V/s) | 61.6 | 9.6 | 10 | 103.1* | 18.4 | 5 |
|  | Repolarization rate (V/s) | -37.9 | 8.1 | 10 | -71.6* | 14.1 | 5 |
|  | Half width (ms) | 2.8 | 0.5 | 10 | 1.7 | 0.2 | 5 |
|  | I Na max (pA/pF) | -84.5 | 18.0 | 10 | -191.3* | 61.8 | 5 |
|  | I K max (pA/pF) | 84.2 | 17.5 | 10 | 167.6 | 56.2 | 5 |

**Table S4. Differential gene expression analysis in the cortex of 2-month old *Neat1^-/-^* vs *Neat1^+/+^* mice.**

*Available as an Excel file.*

**Table S5. Analysis of alternative splicing changes in the cortex of 2-month old *Neat1^-/-^* vs *Neat1^+/+^* mice.**

*Available as an Excel file.*
